# Development and validation of a four‐lipid metabolism gene signature for diagnosis of pancreatic cancer

**DOI:** 10.1002/2211-5463.13074

**Published:** 2021-09-20

**Authors:** Yanrong Ye, Zhe Chen, Yun Shen, Yan Qin, Hao Wang

**Affiliations:** ^1^ Department of Pharmacy Zhongshan Hospital Fudan University Shanghai China; ^2^ Department of Pharmacy Xiamen Branch Zhongshan Hospital Fudan University Xiamen China; ^3^ Teaching Center of Experimental Medicine Shanghai Medical College Fudan University Shanghai China

**Keywords:** four‐gene signature, pancreatic cancer, prognosis, TCGA, WGCNA

## Abstract

Abnormal lipid metabolism is closely related to the malignant biological behavior of tumor cells. Such abnormal lipid metabolism provides energy for rapid proliferation, and certain genes related to lipid metabolism encode important components of tumor signaling pathways. In this study, we analyzed pancreatic cancer datasets from The Cancer Genome Atlas and searched for prognostic genes related to lipid metabolism in the Molecular Signature Database. A risk score model was built and verified using the GSE57495 dataset and International Cancer Genome Consortium dataset. Four molecular subtypes and 4249 differentially expressed genes (DEGs) were identified. The DEGs obtained by Weighted Gene Coexpression Network Construction analysis were intersected with 4249 DEGs to obtain a total of 1340 DEGs. The final prognosis model included *CA8*, *CEP55*, *GNB3* and *SGSM2*, and these had a significant effect on overall survival. The area under the curve at 1, 3 and 5 years was 0.72, 0.79 and 0.87, respectively. These same results were obtained using the validation cohort. Survival analysis data showed that the model could stratify the prognosis of patients with different clinical characteristics, and the model has clinical independence. Functional analysis indicated that the model is associated with multiple cancer‐related pathways. Compared with published models, our model has a higher C‐index and greater risk value. In summary, this four‐gene signature is an independent risk factor for pancreatic cancer survival and may be an effective prognostic indicator.

## Graphical abstract

Abnormal lipid metabolism is closely related to the malignant biological behavior of tumor cells. In this study, we analyzed pancreatic cancer datasets from The Cancer Genome Atlas and searched for prognostic genes related to lipid metabolism in the Molecular Signature Database. Compared with published models, our model has a higher C‐index and greater risk value. This four‐gene signature is an independent risk factor for pancreatic cancer survival and may be an effective prognostic indicator.

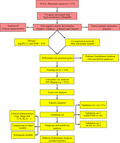

**Abbreviations**
AUC: area under the receiver operating characteristic curve
CI: confidence interval
DEG: differentially expressed gene
FDR: false discovery rate
GEO: Gene Expression Omnibus
GO: Gene Ontology
GSVA: Gene Set Variation Analysis
HR: hazard ratio
ICGC: International Cancer Genome Consortium
KEGG: Kyoto Encyclopedia of Genes and Genomes
KM: Kaplan–Meier
Lasso: least absolute shrinkage and selection operator
NMF: non‐negative matrix clustering algorithm
OS: overall survival
RNA‐seq: RNA sequencing
ROC: receiver operating characteristic
ssGSEA: single‐sample gene set enrichment analysis
TCGA: The Cancer Genome Atlas
TIMER: Tumor Immune Estimation Resource
WGCNA: Weighted Gene Coexpression Network Construction

As a malignancy of the digestive system, pancreatic cancer is one of the most aggressive malignancies in the world. In recent years, pancreatic cancer morbidity and mortality rates have steadily increased, with an overall 5‐year survival rate of about 8% for patients [1]. Despite the rapid development of diagnosis and treatment of pancreatic cancer over the past 20 years, the mortality rate of patients remains high [2, 3, 4]. The combination of tumor markers and imaging helps to diagnose the disease in a timely and accurate manner [5]; however, due to the insidious nature of the disease and the lack of early clinical signs, it is not easy to diagnose. It has been reported that about 50% of patients have confirmed that the cancer has metastasized [6, 7]. Therefore, revealing the molecular mechanisms of pancreatic cancer progression and developing corresponding targeted therapies are critical to improving pancreatic cancer outcomes.

Lipids play an important function in maintaining normal cell function and homeostasis; they are not only an important part of the cell membrane but also provide precursors for important molecules needed in the growth and differentiation pathways [8, 9]. Intracellular lipids come from two sources: one is food intake, and the other is lipid synthesis from scratch by hepatocytes and cells in need. Normal cells in the body acquire lipids mainly from diet and rarely from lipid synthesis from scratch. For most normal cells, lipids that meet cellular needs rarely are synthesized from scratch because of slow cell growth [10, 11]. However, Medes *et al*. [12] found in the 1950s that tumor cells synthesize fatty acids primarily by synthesizing them from scratch. In tumor cells, on the one hand, lipids and cholesterol are often activated to meet the needs of tumor cells that are rapidly proliferating, and on the other hand, lipids alter the properties of biofilms and protect cells from oxidative damage from internal and external sources [13]. Lipogenesis is an important feature of rapid malignancy growth [14]. Normal cell lipid synthesis from scratch is rare, and about 90% of fatty acids are synthesized from scratch in tumor cells [12]. Activated lipid scratch synthesis was found to be associated with poorer prognosis and shorter disease‐free survival in tumor patients [15, 16]. At the molecular level, increased lipid synthesis from scratch in tumors is often accompanied by increased lipid synthase and enhanced activity [17, 18]. Thus, aberrant activation of lipid synthesis from scratch is a common feature of tumor cells. In addition, lipid metabolic reprogramming that promotes increased lipogenesis is associated with the abnormal development and progression of pancreatic adenocarcinoma [19]. Mammalian target of rapamycin complex 2 (mTORC2) stimulates the synthesis of sphingomyelin (glucoceramide) and glycerophospholipids (cardiolipin) to promote tumor progression [20]. Studies have shown that mTORC1 stimulates the synthesis of fatty acids and sterols by regulating the expression of SREBP1c, a major adipogenic transcription factor [20, 21, 22, 23]. The active form of SREBP1c is sensitive to proteasomal degradation but can enter the nucleus and participate in its transcriptional targets, including its own gene promoter and the promoter of the major enzyme encoding fatty acid synthesis [24]. However, a deeper understanding of lipid metabolism‐related genes in the prognosis and treatment of pancreatic cancer is needed.

In this study, lipid metabolism‐related gene expression in pancreatic cancer was analyzed to identify key genes that could predict patient prognosis. A differentially expressed gene (DEG) analysis, Weighted Gene Coexpression Network Construction Analysis (WGCNA) and Cox proportional risk model were used to finally construct a signature based on the expression of several key genes as a prognostic signature for pancreatic cancer. This prognostic model can be used as an effective tool to predict the prognosis of patients with pancreatic cancer. These findings will also help identify new therapeutic targets for pancreatic cancer.

## Material and methods

### Expression spectral data and preprocessing

**Fig. 1:**
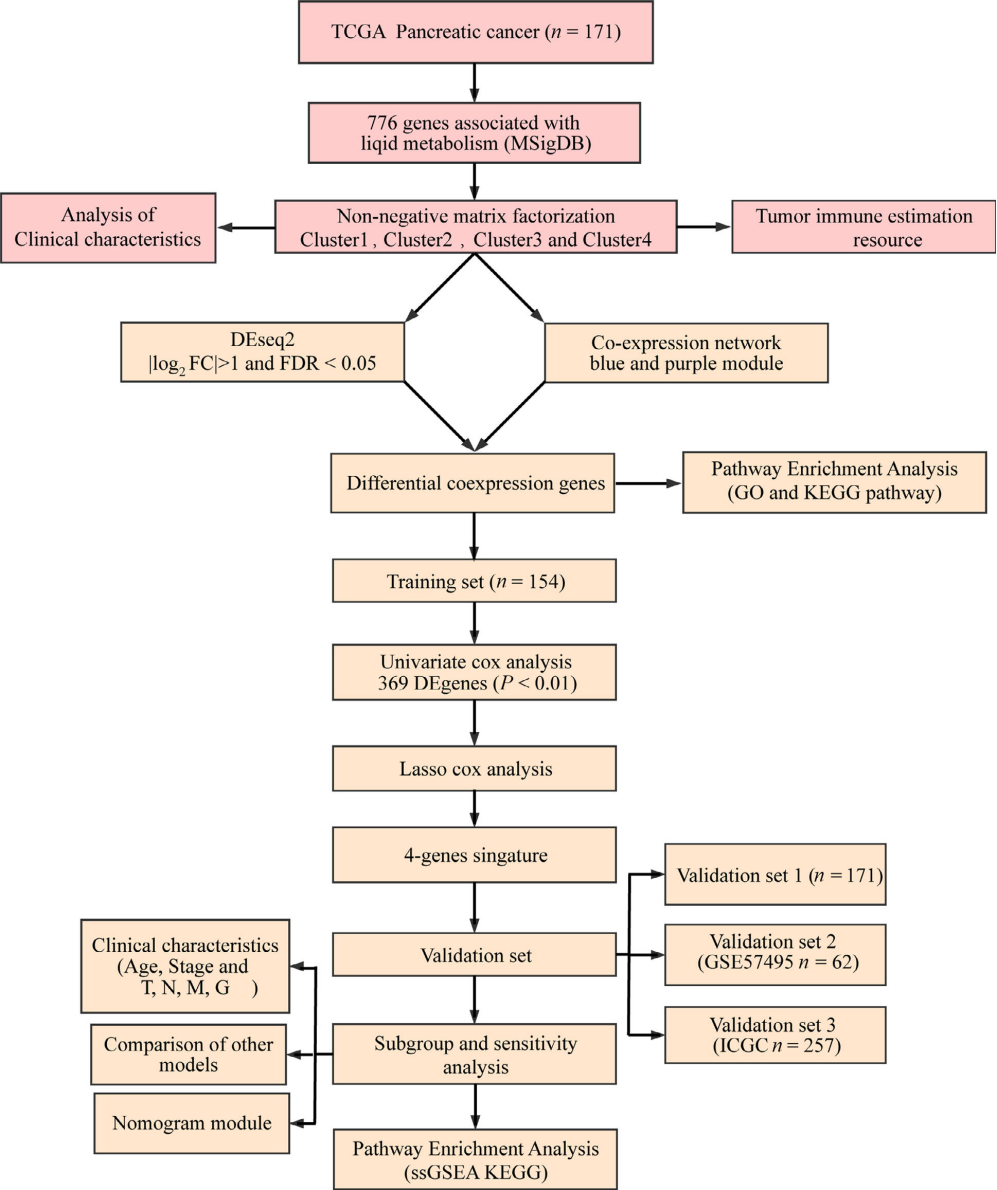
Workflow chart. FC, fold change.

Human lipid metabolics‐related pathways were downloaded from Molecular Signature Database v7.0 [25], and a total of 776 genes related to lipid metabolism were sorted out from the six lipid metabolic pathways in Table 1. Pancreatic cancer RNA sequencing (RNA‐seq) expression data and corresponding clinical follow‐up data were obtained from the public database The Cancer Genome Atlas (TCGA) (https://portal.gdc.cancer.gov/) [26], which contained RNA‐seq data of 182 patients and clinical information of 171 patients on December 3, 2019. GSE57495 is a microarray dataset from the Gene Expression Omnibus (GEO) database (http://www.ncbi.nlm.nih.gov/geo/) [27], containing expression profile data and clinical sample information from 62 patients with early pancreatic cancer. The International Cancer Genome Consortium (ICGC) validation dataset included 257 patients with pancreatic cancer with expression profile data and clinical follow‐up information. For TCGA dataset, (a) samples without clinical data and overall survival (OS) <30 days were removed, (b) normal tissue sample data were removed, (c) genes with fragments per kilobase of exon per million of zero in half of the samples were removed, and (d) the expression profile of genes related to lipid metabolism was preserved. For GEO datasets, (a) normal tissue sample data were removed, (b) OS data from months was converted to days, (c) samples with OS <30 days were removed, (d) Chip probes were mapped to the human gene SYMBOL using Bioconductor package, and (e) the expression profile of genes related to lipid metabolism was preserved. For the ICGC dataset, (a) sample data without survival status were removed, (b) samples with OS <30 days were removed, and (c) the expression profile of genes related to lipid metabolism was preserved. The GSE57495 and ICGC datasets were considered as the validation dataset. The clinical data information is shown in Table 2. The workflow chart is shown in Fig. 1.

**Table 1:** Pathways involved in lipid metabolism.

Pathways	Database	Gene count
Regulation of lipid metabolism by peroxisome proliferator‐activated receptor alpha	Reactome	119
Metabolism of lipids	Reactome	738
Sphingolipid metabolism	Reactome	89
Transcriptional regulation of white adipocyte differentiation	Reactome	84
Glycerophospholipid metabolism	KEGG	77
Fatty acid metabolism	Reactome	177
Sum		1,284 (unique: 776)

**Table 2:** The clinical information of four datasets.

Characteristics	TCGA set	Training set	*P* value	GSE57495 set	ICGC set
Age (years)					
<65	78	71	1.00	–	103
≥65	93	83		–	154
Progression‐free survival					
Alive	80	69	1.00	21	151
Dead	91	85		41	106
Sex					
Female	78	72	1.00	–	120
Male	93	82		–	137
pathologic_T					
T1	7	7	0.09	–	–
T2	21	19		–	–
T3	138	123		–	–
T4/TX	4	4		–	–
pathologic_N					
N1	119	106	0.21	–	–
N0/NX	51	47		–	–
pathologic_M					
MX	90	81	0.199	–	–
M0/M1	81	72		–	–
Tumor stage					
Stage I	19	18	0.125	–	–
Stage II	142	127		–	–
Stage III	3	3		–	–
Stage IV	3	3		–	–
pathologic_G					
G1	28	33324	0.241	–	–
G2	92	84		–	–
G3	47	43		–	–
G4	2	2		–	–
Total	171	154	–	62	257

### Identification of prognostic genes

The expression profile of 776 lipid metabolism genes was extracted from TCGA dataset. However, 15 genes were not found. Furthermore, we keep genes that are not zero in more than half of the samples. As a result, 730 genes were used for subsequent analysis. Next, univariate Cox analysis of coxph function in r package was performed to obtain genes related to prognosis of pancreatic cancer with *P* < 0.05.

### Identification of molecular subtypes

Cluster analysis of pancreatic cancer samples was performed by nonnegative matrix clustering algorithm (NMF), and the standard ‘brunet’ was selected by NMF method for 50 iterations. The clustering number *k* was set as 2–10, and the average contour width of the common member matrix was determined through the r package ‘NMF’. The minimum member of each subclass was set as 10. According to the cophenetic, dispersion and silhouette index were used to determine the optimal clustering number.

### Difference of tumor‐infiltrating immune cells in molecular subtypes

Six types (B_cell, CD4_Tcell, CD8_Tcell, neutrophil, macrophage cell and dendritic cell) of tumor‐infiltrating immune cell were retrieved from Tumor Immune Estimation Resource (https://cistrome.shinyapps.io/timer/) [28]. Immunity, matrix score, and tumor purity of each sample were calculated in r package estimate. These indicators were compared on molecular subtypes.

### Identification of DEGs

r package differentially expressed Seq2 (DESeq2) [29] was applied to calculate the DEGs in molecular subtypes with a false discovery rate (FDR) <0.05 and |log_2_FC| > 1.

### WGCNA

Based on expression profiles of DEG, the WGCNA coexpression algorithm was used to mine the coexpression module using the r package WGCNA (http://www.r‐project.org/) [30]. First, the appropriate soft threshold is determined by approximate scale‐free topology criteria. The adjacency matrix was transformed into a topological matrix, and the genes were clustered using average‐linkage hierarchical clustering. Lastly, the dynamic tree cut method was used to determine module eigengenes, at least 30 coexpressed genes.

### Functional enrichment and pathway enrichment analysis

Gene Ontology (GO) enrichment analysis and Kyoto Encyclopedia of Genes and Genomes (KEGG) pathway analysis were performed for DEG based on ‘WebGestaltR’ [31] in r. FDR < 0.05 was defined as significant.

### Construction of a prognostic risk model based on differentially coexpressed genes

First, 90% of samples were randomly selected from the preprocessed 171 TCGA samples as the training set for model construction. To avoid the random allocation bias affecting the stability of subsequent modeling, we repeatedly sampled 100 samples with replacement in advance to ensure that the randomly selected samples were consistent with all samples in age, stage and TNM staging. Univariate Cox regression analysis for OS was performed to identify prognostic DEGs with *P* < 0.05 using survival coxph function in r. Lasso (least absolute shrinkage and selection operator) Cox regression analysis was performed to find characteristic genes using r package glmnet. Subsequently, the multivariate Cox proportional hazards regression model was used to build a prognostic model in the training group. The risk formula was as follows: RiskScore_4 _= −0.0666 × *CA8* + 0.0413 × *CEP55* − 0.2189 × *GNB3* − 0.0339 × *SGSM2*. Next, the Kaplan–Meier (KM) survival curve was used to compare prognosis between the low‐ and high‐risk groups, which were classified by the median risk score as the cutoff value in all patients. The receiver operating characteristic (ROC) curve was applied to assess diagnostic accuracy through comparing the areas under the ROC curves (AUCs) using timeROC package in the training and validation groups.

### Gene set enrichment analysis

The r software package Gene Set Variation Analysis (GSVA) [32] was used for single‐sample gene set enrichment analysis (ssGSEA), and the function with correlation >0.45 was selected.

### Advantages of genetic signatures

To identify the independence of four gene signatures, we used univariate and multivariate Cox regression to analyze the relationship among age, sex, pathological stage T, stage N, stage M, tumor stage, grade and RiskScore with prognosis. Next, by referring to the literature, we selected four prognostic risk models, 15‐gene signature (Chen) [33], 7‐gene signature (Cheng) [34], 5‐gene signature (Raman) [35] and 9‐gene signature (Wu) [36], for comparison with our 4‐gene model. ROC curve and KM survival curve of four models were drawn in TCGA dataset. Furthermore, we compared the four models with the restricted mean survival (RMS) using r language RMS [37] and standardized net benefit between four models and four gene signatures.

## Results

### Identification of four molecular subtypes of pancreatic cancer

**Fig. 2:**
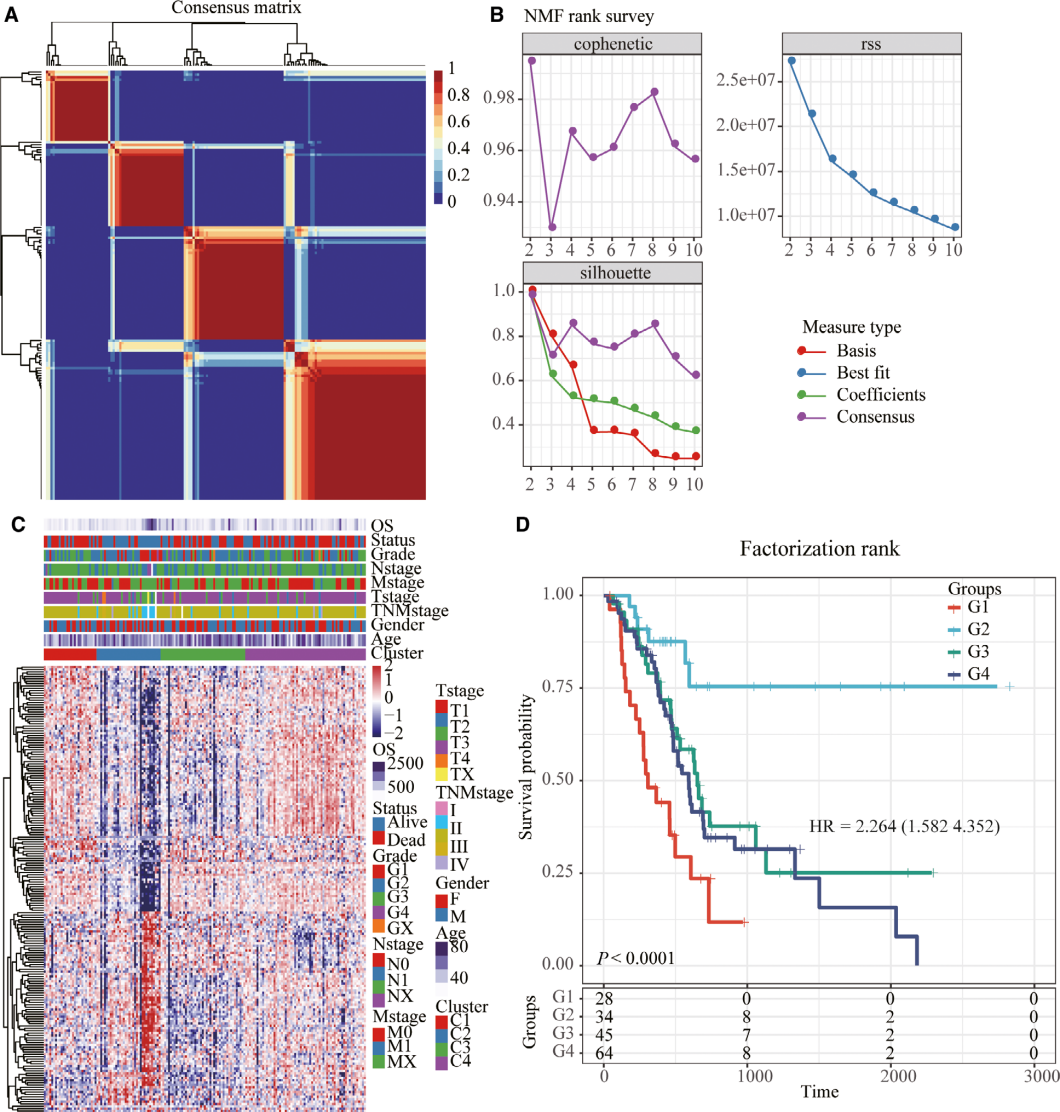
Identification of molecular subtypes of pancreatic cancer. (A) Consensus map of NMF clustering. (B) The distribution of cophenetic, rss and dispersion with rank = 2–1. (C) Cluster heatmap of 740 lipid metabolism genes. (D) KM prognostic survival curve of molecular subtype.

Univariate Cox survival analysis of lipid metabolism genes using coxph revealed 189 genes associated with the prognosis of pancreatic cancer. Pancreatic cancer samples were clustered by NMF algorithm, and the optimal number of clusters was determined to be four based on cophenetic, dispersion and silhouette metrics (Fig. 2A,B). The expression of lipid metabolism‐related genes showed that the expression of C2 genes was lower than that of C1, C3 and C4 genes (Fig. 2C). Analysis of the prognostic relationship among the four subtypes showed that C1 had the worst prognosis and a significant difference [*P* < 0.0001; hazard ratio (HR), 2.264 (1.582–4.352)], and C2 had the best prognosis (Figs 2D and S1). Subsequently, we compared the clinical characteristics of the four subtypes and found significant differences in T, TNM, cancer grade and age (Fig. S2). The immune evaluation of the four subtypes showed significant differences in immune scores and stroma (Fig. S3).

### Identification of differential expression genes and functional analysis

**Fig. 3:**
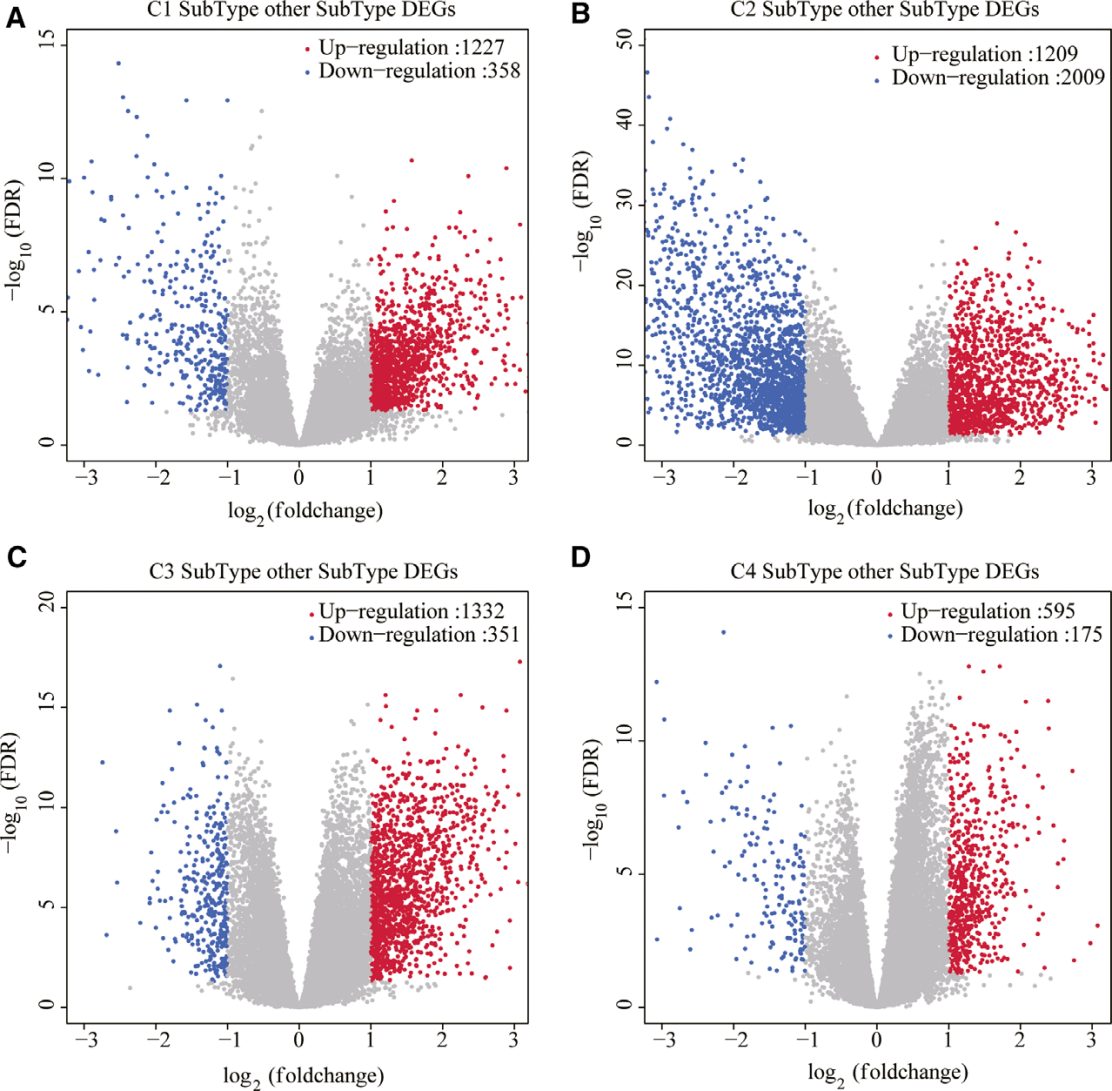
Identification of DEG. (A) Volcano diagram of DEG in C1 subtype. (B) Volcano diagram of DEG in C2 subtype. (C) Volcano diagram of DEG in C3 subtype. (D) Volcano diagram of DEG in C4 subtype.

**Fig. 4:**
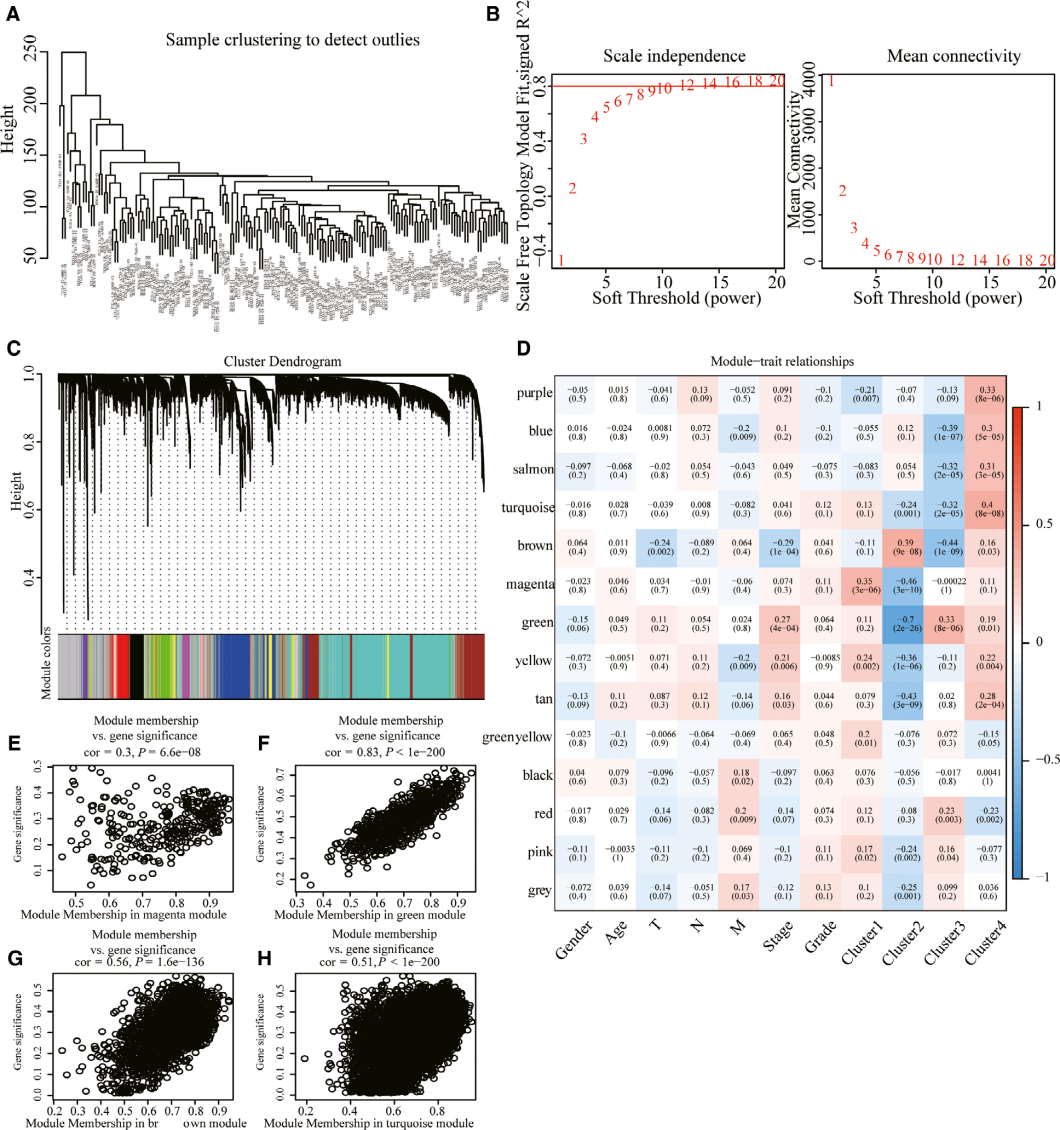
WGCNA. (A) Cluster analysis of samples. (B) Analysis of network topology for various soft‐thresholding powers. (C) Gene dendrogram and module colors. (D) Correlation between 14 modules and clinical phenotype. (E) Gene significance (*y* axis) versus module membership (*x* axis) plotted for magenta module in TCGA dataset. (F) Gene significance (*y* axis) versus module membership (*x* axis) plotted for green module in TCGA dataset. (G) Gene significance (*y* axis) versus module membership (*x* axis) plotted for brown module in TCGA dataset. (H) Gene significance (*y* axis) versus module membership (*x* axis) plotted for turquoise module in TCGA dataset.

**Fig. 5:**
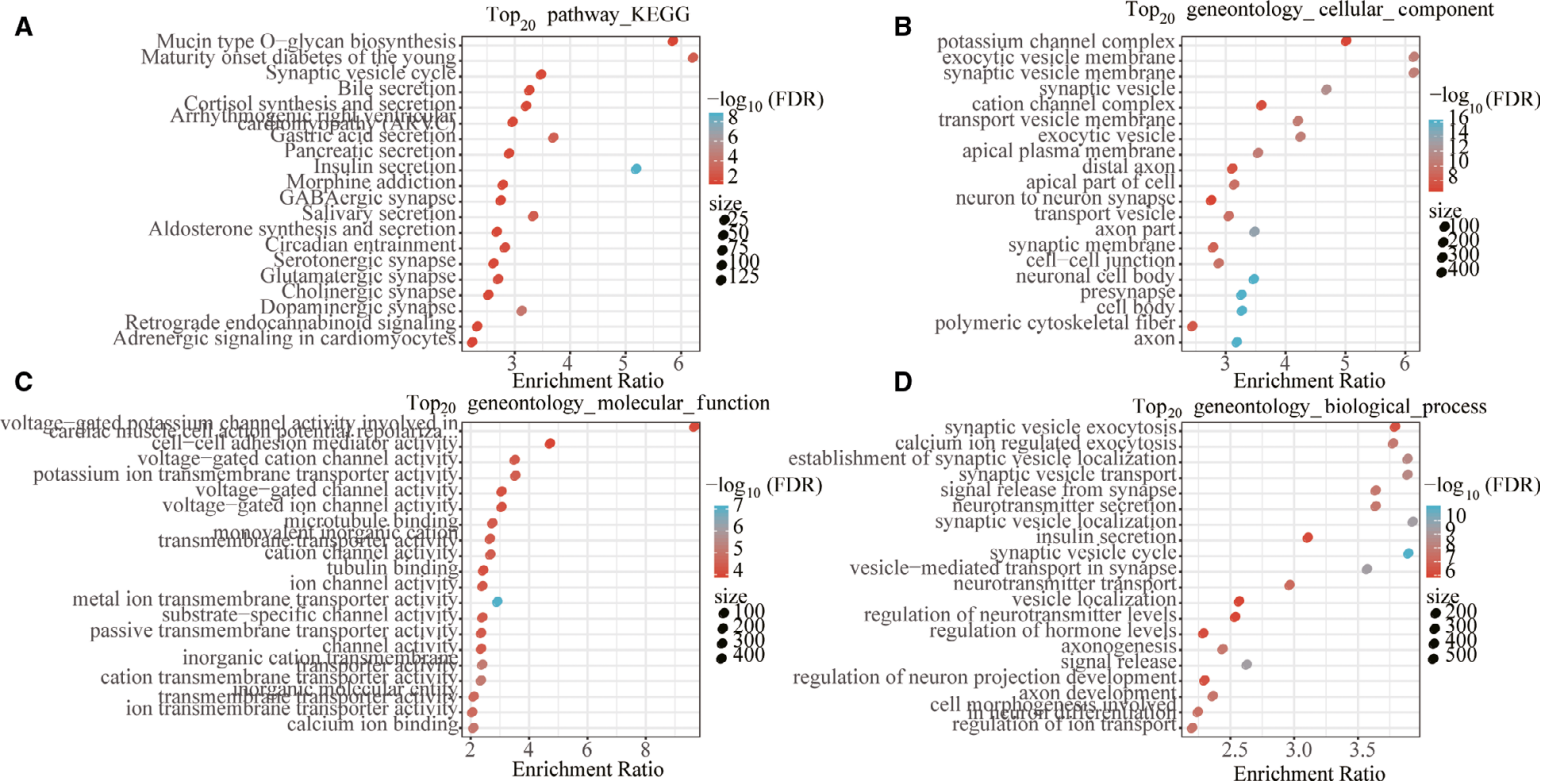
Functional analysis of differentially coexpressed genes. (A) The DEGs were enriched to the top 20 enriched by KEGG. (B) The DEGs were enriched to the top 20 enriched by GO CC. (C) The DEGs were enriched to the top 20 enriched by GO MF. (D) The DEGs were enriched to the top 20 enriched by GO BP. The color from red to blue represents the significance of *P* value; the darker the red the smaller is the *P* value, and the dot size represents the number of genes enriched into the pathway (the higher the number, the larger the dot).

DESeq2 was conducted to calculate the DEGs between C1 and C2, C3 and C4 molecular subtypes; a total of 4249 were obtained (Fig. 3). Next, according to the expression profile of coding genes, the WGCNA coexpression algorithm was used to mine the coexpressed coding genes and coexpression modules, and hierarchical clustering analysis was performed on the samples to show that there were no outlier samples (Fig. 4A). A soft threshold of 10 was selected (Fig. 4B,C). Genes were clustered using the averages‐linkage hierarchy clustering method, and 14 modules were obtained by setting height = 0.25, deepSplit = 2 and minModuleSize = 30 (Fig. 4D). The correlation of each module with sex, age, ethnicity and clusters 1, 2, 3 and 4 was further analyzed. The results showed that the modules significantly related to clusters 1, 2, 3 and 4 were magenta, green, brown and turquoise, respectively (Fig. 4E–H). Finally, 4249 DEGs and genes with significant coexpression modules were intersected to obtain 1340 differentially expressed cogenes. To determine whether differential genes are related to pancreatic cancer function, we performed GO molecular function and KEGG function enrichment analysis of 1340 differential genes using r software package WebGestaltR (https://www.r‐project.org/help.html). The significant pathways enriched by KEGG are related to insulin secretion and dopaminergic synapse pathways (Fig. 5A). GO enriched 233 GO cellular component (CC) (Fig. 5B), 195 GO molecular function (MF) (Fig. 5C) and 977 GO biological process (BP) (Fig. 5D).

### Construction and risk prediction of Gene Signature four‐gene signature

**Fig. 6:**
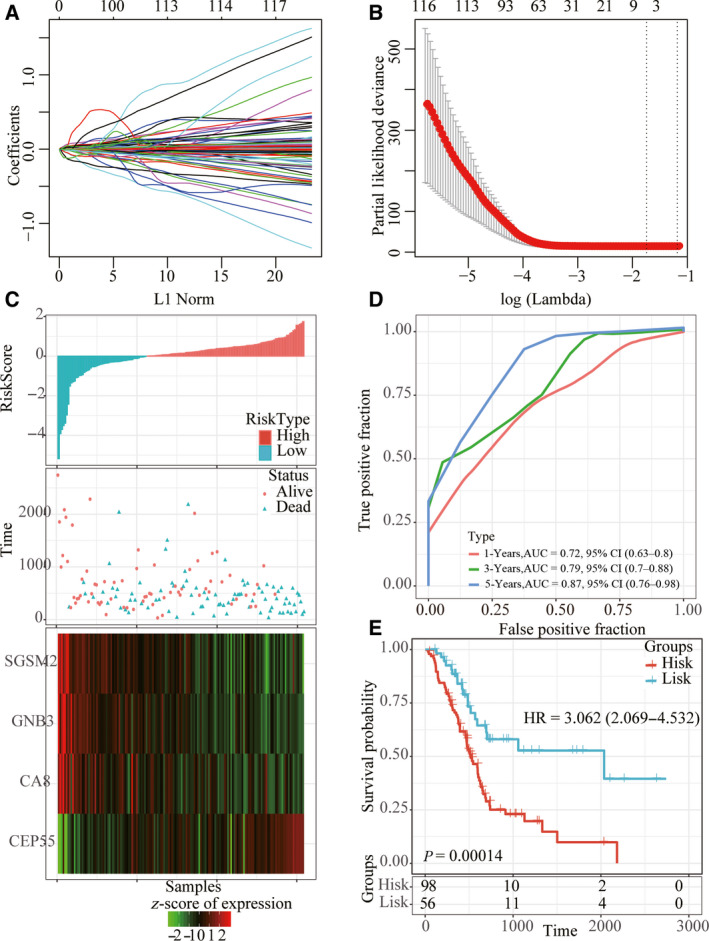
Construction of risk model. (A) The CI for each lambda. (B) The trajectory of each independent variable, the log of lambda on the horizontal axis and the coefficient on the vertical axis. (C) Risk score, survival time and survival status, and expression of four genes in the training set. (D) ROC curve and AUC of four‐gene signature in the training set. (E) KM survival curve of four‐gene signature in the training set.

Ninety percent of the 171 TCGA samples were randomly selected as the training set for model construction. Univariate Cox proportional hazard analysis was conducted for the expression profile of each differentially expressed cogene, and r package survival coxph function was used to obtain 369 genes with significant prognostic differences. To further narrow the range of genes and construct the prognostic model with high accuracy, we used r software package glmnet for Lasso Cox regression analysis. First, the analysis of the change trajectory of each independent variable shows that as the lambda gradually increases, the number of independent variable coefficients tending to zero also gradually increases (Fig. 6A). Then the confidence interval (CI) under each lambda is analyzed, and the model reaches the optimal value when lambda = 0.1753479 (Fig. 6B). Four genes were selected at lambda = 0.1753479 as target genes (Table 3). The four‐mRNA signature formula is as follows:\def\mybox{\vrule depth ‐0.5mm height 4mm width 8mm}

**Table 3:** Four‐mRNA signature.

Symbol	Coefficient	HR	*z*‐score	*P* value	Low 95% CI	High 95% CI
CA8	−0.06663	−2	0.045465	0.9355	0.8764	0.9987
CEP55	0.04133	3.639	0.000274	1.0422	1.0193	1.0657
GNB3	−0.21887	−1.397	0.162309	0.8034	0.591	1.0921
SGSM2	−0.03386	−1.6	0.10963	0.9667	0.9274	1.0077

RiskScore4 = −0.0666 × *CA8* + 0.0413 × *CEP55* − 0.2189 × *GNB3* − 0.0339 × *SGSM2*.

The RiskScore of each sample is calculated according to the expression level of the sample, and the RiskScore of the sample is plotted. The survival time of the samples with high RiskScore was significantly lower than that with low RiskScore. The gene expression changes with the increase of risk value showed that *CEP55* was a risk factor, whereas *CA8*, *GNB3* and *SGSM2* were protective factors (Fig. 6C). ROC analysis of RiskScore by r software package timeROC showed that AUC of 1, 3 and 5 years was >0.70 (Fig. 6D). Finally, we carried out *z*‐score for RiskScore and divided the samples with *z*‐score‐based RiskScore greater than zero into the high‐risk group and the samples with less than zero into the low‐risk group. KM prognostic analysis showed a significant difference between the two groups (Fig. 6E).

### Robustness of four‐gene signature

**Fig. 7:**
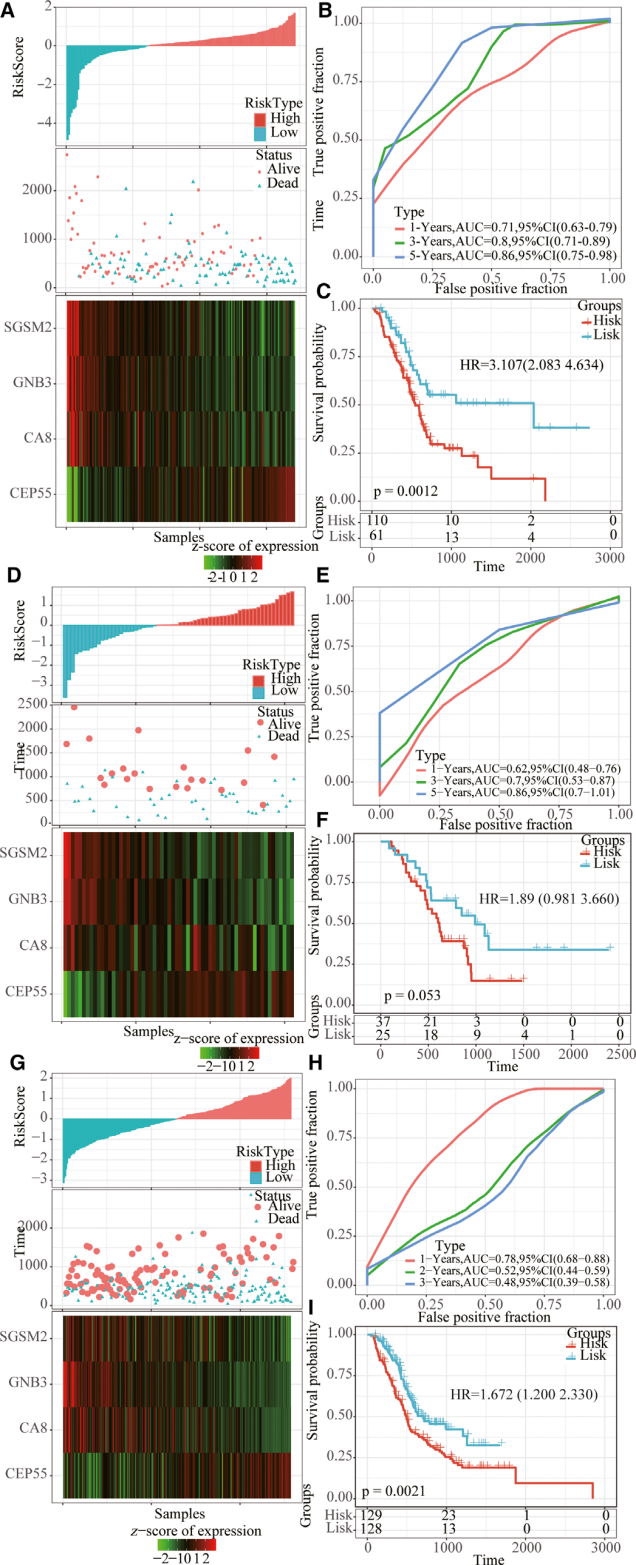
Robustness of risk model. (A, D, G) Risk score, survival time and survival status, and expression of four genes in whole TCGA dataset, GSE57495 dataset and ICGC verification set. (B, E, H) ROC curve and AUC of four‐gene signature in whole TCGA dataset, GSE57495 dataset and ICGC verification set. (C, F, I) KM survival curve of four‐gene signature in whole TCGA dataset, GSE57495 dataset and ICGC verification set.

To determine the robustness of the model, we used the whole TCGA dataset, GSE57495 dataset and ICGC verification set as the verification dataset, and the same model and coefficient as the training set were adopted. Similarly, the high RiskScore sample had a worse prognostic capability, *CEP55* was a risk factor, and *CA8*, *GNB3* and *SGSM2* were protective factors (Fig. 7A,D,G). ROC analysis showed that the model had high AUC (Fig. 7B,E,H). The results of the KM curve showed that there were significant marginal differences between the two groups (Fig. 7C,F,I). Moreover, we obtained three additional pancreatic cancer datasets from the GEO database, GSE28735, GSE62452, and GSE85916, and we used the same method to score the risk for each patient in these three cohorts. First, we performed ROC analysis on the GSE28735 dataset. Because of the short follow‐up period, it was not possible to calculate the 5‐year AUC, in which the AUC of 1 and 3 years reached more than 0.78 (Fig. S4A), and there was a significant prognostic difference between the high‐ and low‐risk groups (Fig. S4D). ROC analysis in the GSE62452 dataset showed the highest AUC of 0.77 for 3 years (Fig. S4B), with significant prognostic differences between the high‐ and low‐risk groups (Fig. S4E). ROC analysis in the GSE85916 dataset showed the highest 5‐year AUC of 0.84 (Fig. S4C), with a significant prognostic difference between the high‐ and low‐risk groups (Fig. S4F).

### Prognostic analysis of risk models and clinical features

**Fig. 8:**
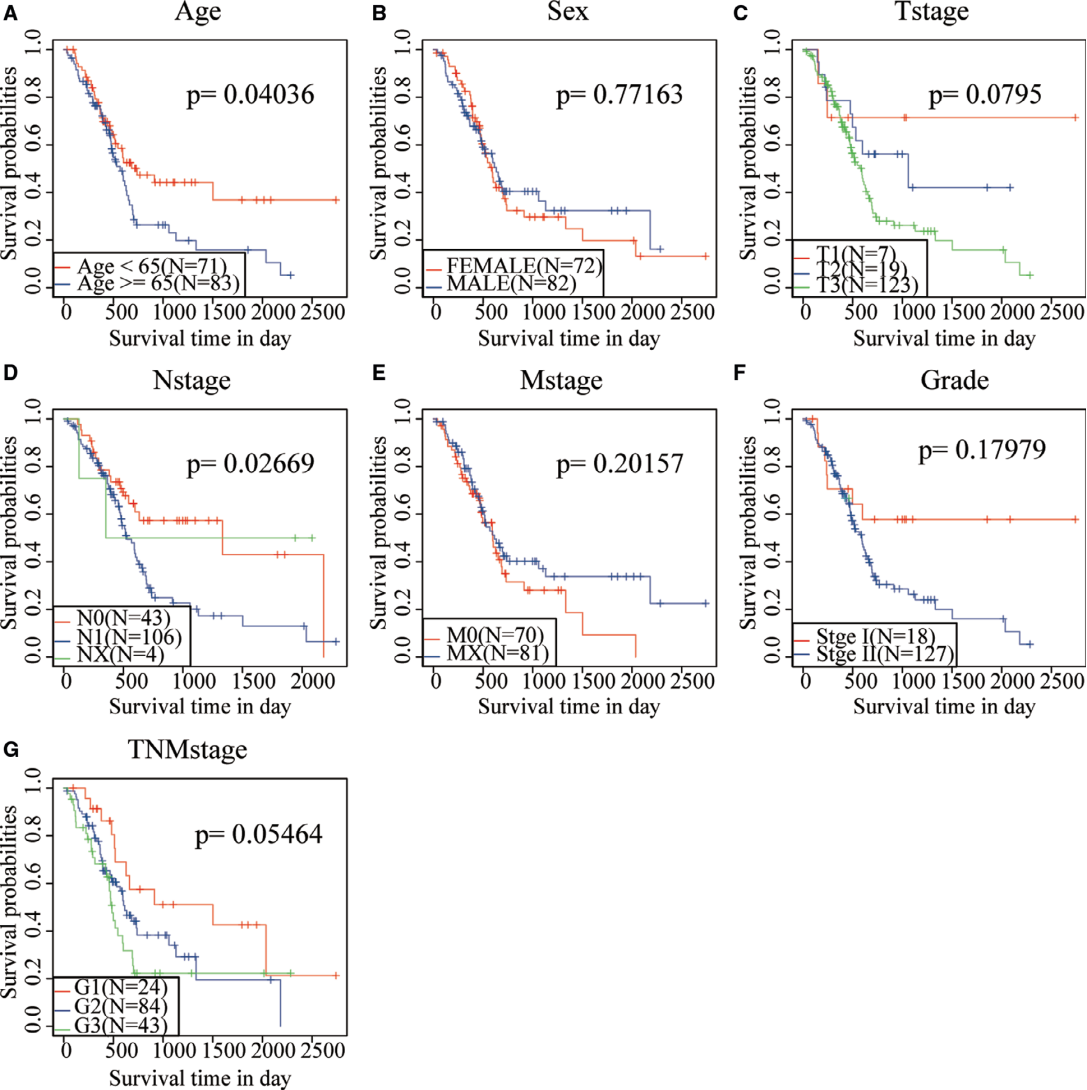
Prognostic analysis of clinical characteristics and risk models. (A) KM prognosis curve in age samples. (B) KM prognosis curve in sex samples. (C) KM prognosis curve in T stage samples. (D) KM prognosis curve in N stage samples. (E) KM prognosis curve in M stage samples. (F) KM prognosis curve in grade samples. (G) KM prognosis curve in TNM stage samples.

**Fig. 9:**
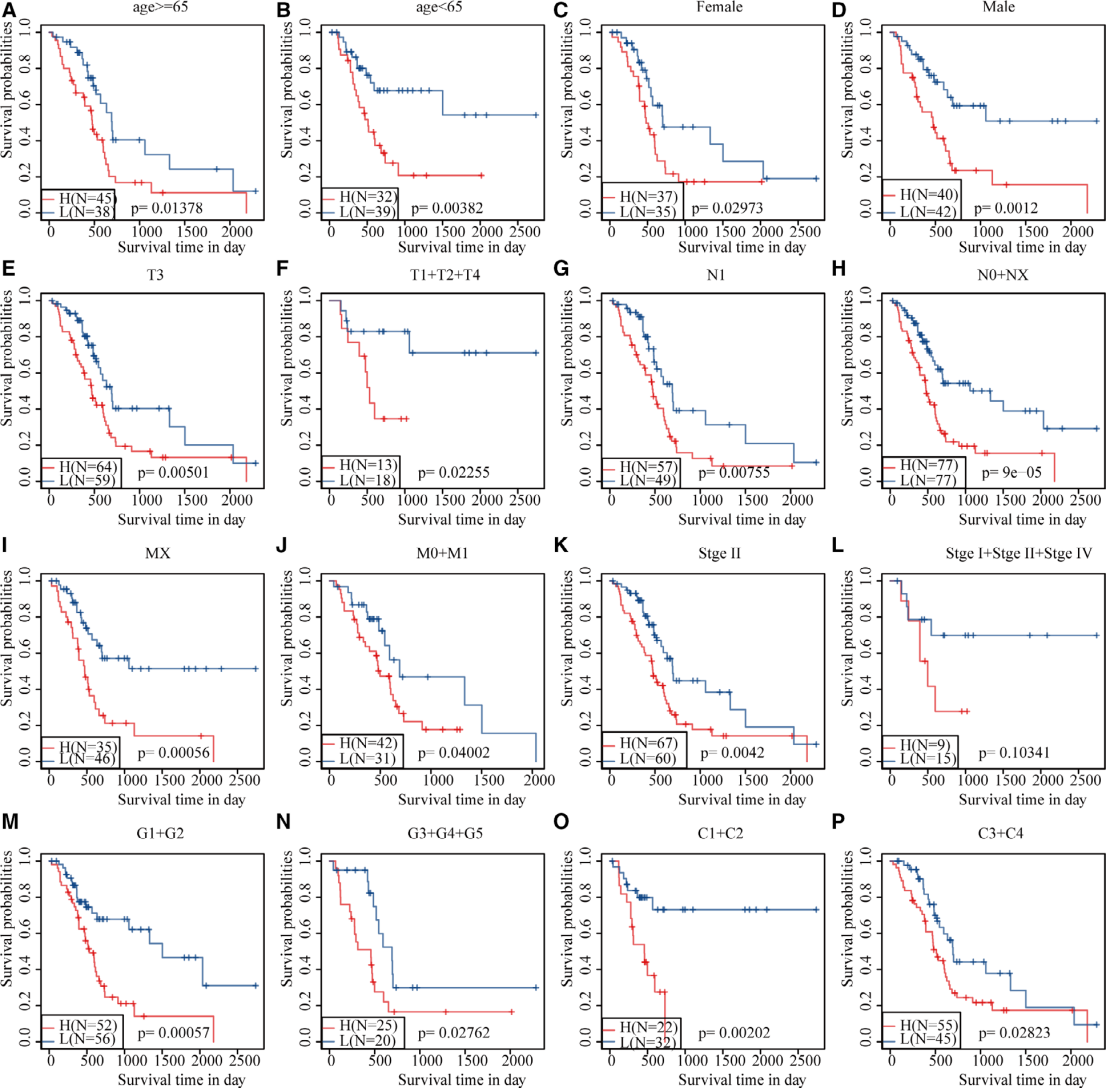
Prognostic analysis of clinical characteristics and risk models. (A) KM prognosis curve in young samples (age ≤ 65 years). (B) KM prognosis curve in old samples (age > 65 years). (C) KM prognosis curve in female samples. (D) KM prognosis curve in male samples. (E) KM prognosis curve in T3 stage samples. (F) KM prognosis curve in T1 + T2 + T4 stage samples. (G) KM prognosis curve in N1 stage samples. (H) KM prognosis curve in N0 + NX stage samples. (I) KM prognosis curve in MX stage samples. (J) KM prognosis curve in M0 + M1 stage samples. (K) KM prognosis curve in stage II samples. (L) KM prognosis curve in stage I + II + IV samples. (M) KM prognosis curve in G1 + G2 stage samples. (N) KM prognosis curve in G3 + G4 + G5 stage samples. (O) KM prognosis curve in C1 + C2 samples. (P) KM prognosis curve in C3 + C4 samples.

Survival analysis showed that only age, N stage and OS were significantly correlated in the TCGA training set sample (*P* < 0.05), and TNM stage presented significant margin (*P* = 0.05464; Fig. 8). It was further found that four‐mRNA signature could distinguish the young and old groups, female, stage I + II, T1 + T2 and T3 + T4 patients from high‐ and low‐risk groups (*P* < 0.05; Fig. 9). These data further illustrate that our model still has good predictive ability in different clinical signs.

### Clinical independence and regulatory pathway of four‐mRNA signature

**Fig. 10:**
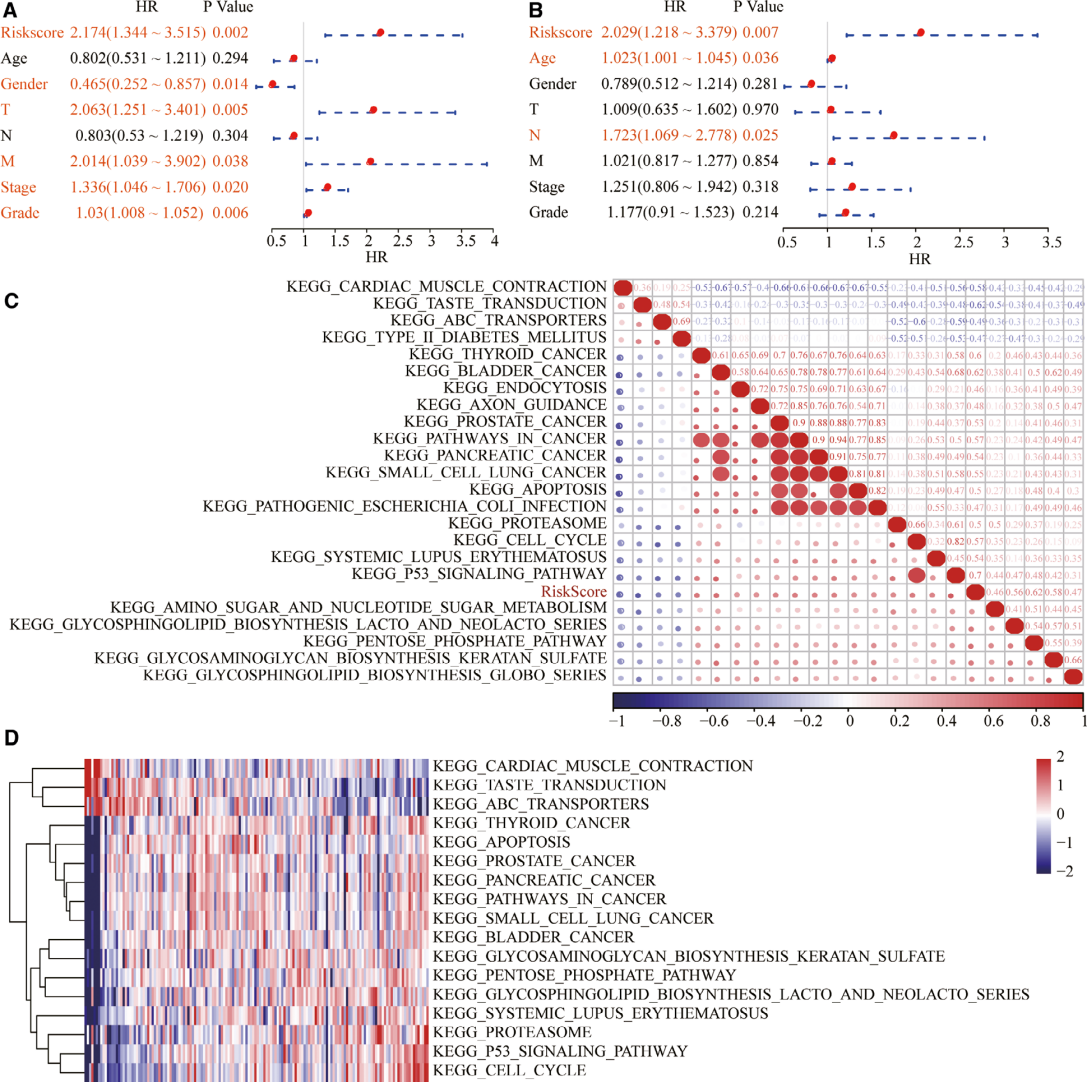
Clinical independence and regulatory pathway of four‐mRNA signature. (A) Univariate Cox regression analysis of four‐mRNA signature. (B) Multivariate Cox regression analysis of four‐mRNA signature. (C) Clustering of correlation coefficients between KEGG pathways and RiskScore with a correlation >0.45. (D) The change of ssGSEA score in each sample with the increase of risk score in the KEGG pathway. The horizontal axis represents the sample, and the risk score increases from left to right.

To identify the independence of the four‐mRNA signature model in clinical applications, we used univariate and multivariate Cox regression analysis to analyze relevant HR, 95% CI of HR and *P* value in the clinical information carried by the whole TCGA data. In TCGA dataset, the univariate COX regression analysis found that sex, T3, T4 versus T1/T2, stage III versus stage I, II and IV, and RiskScore are significantly associated with survival, but the corresponding multivariate Cox regression analysis found that age, stage of N and risk score (HR, 3.606; 95% CI: 1.659–7.839; *P* = 0.007) were significantly associated with survival (Fig. 10A,B). The earlier conditions indicate that our model four‐mRNA signature has good predictive performance in clinical application value. To observe the relationship between risk scores of different samples and biological function, we used the r software package GSVA for ssGSEA analysis. The function with a correlation >0.45 was selected, from which it can be seen that most of them are negatively correlated with the risk score of the sample, while a few are positively correlated with the risk score of the sample (Fig. 10C). Cluster analysis results showed that among the 17 pathways, KEGG_P53_SIGNALING_PATHWAY, KEGG_SYSTEMIC_LUPUS_ERYTHE MATOSUS, KEGG_CELL_CYCLE and other metabolic‐related pathways increased with the increase of RiskScore, and KEGG_pentose_phosphoate_pathway declined with the increase of RiskScore (Fig. 10D). This also suggests that the dysfunction of these pathways is closely related to tumor development.

### Advantages of risk models

**Fig. 11:**
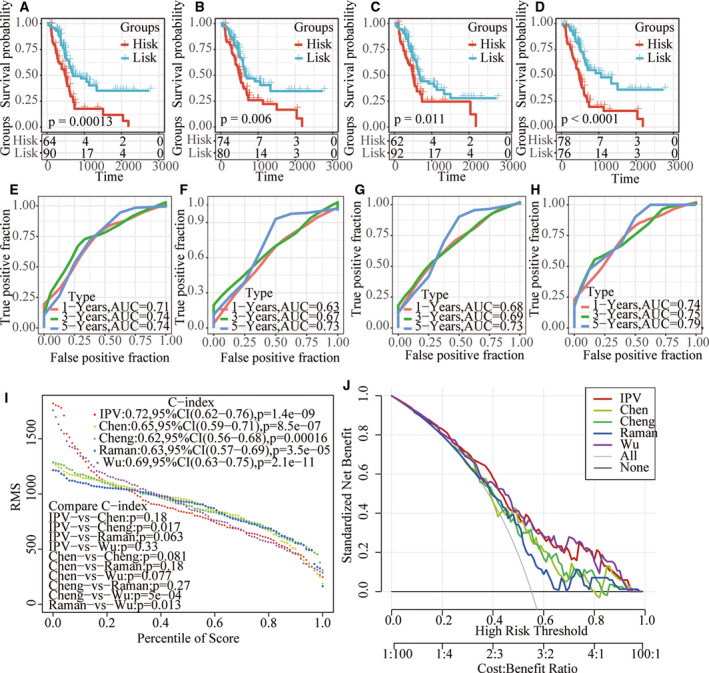
The superiority of the risk model. (A–D) KM prognosis curve of four models. (E–H) AUC of 1, 3, 5 and 3 years in four models. (I) RMS curves of five models. (J) Risk coefficient curves of five models.

Four prognosis‐related risk models, 15‐gene signature (Chen), 7‐gene signature (Cheng), 5‐gene signature (Raman) and 9‐gene signature (Wu), were selected and compared with our four‐gene model. To make the models comparable, we calculated the risk scores of each pancreatic cancer sample in TCGA using the same method. Samples were divided into the risk‐H and risk‐L groups according to the median risk score, and the KM prognosis curve showed that there were significant differences in OS prognosis of samples from the four models in the risk‐H and risk‐L groups (*P* < 0.05; Fig. 11A–D). The ROC analysis results of the model showed that the prediction effect of the four models was worse than that of the four‐gene signature models (Fig. 11E–H). The RMS curve was further drawn using the r language RMS package, indicating that the AUC of the four gene models was higher than that of the four models (Fig. 11I). Curves of risk coefficients also show that the model is relatively high (Fig. 11J).

## Discussion

Pancreatic cancer is by default the king of all cancers, placing a heavy burden on families and the world [38]. Accurate prediction of the prognosis of pancreatic cancer is important for the choice of treatment and the improvement of the prognosis. In this study, four independent datasets were used to identify DEGs associated with lipid metabolism between pancreatic cancer and normal cervical tissue. A total of 189 DEGs were identified, and four molecular isoforms were identified based on lipid metabolism genes combined with WGCNA to finally identify four gene signatures.

The prognostic value of various gene expression profiles has been studied in pancreatic cancer over the past decade. For example, Demirkol Canli *et al*. [39] identified a gene signature composed of 20 prognostic genes (PPS20), indicating OS and event‐free survival of pancreatic cancer. Wolfe *et al*. [40] developed a four‐miRNA molecular signature that is associated with risk for local‐regional recurrence and OS after pancreatic cancer resection. Meng *et al*. [41] constructed a novel eight‐mRNA signature to predict the prognosis of PAAD patients by applying ESTIMATE scoring to RNA‐seq‐based transcriptome data. Chen *et al*. [33] developed a prognostic 15‐gene signature to know OS by analyzing microarray data from 63 patients with early pancreatic ductal adenocarcinoma (PDAC) (stages IB, IIA and IIB) in the Moffitt cohort. Although these reports are promising, the proposed genome is either too large or traditionally genetically composed. Therefore, focusing on key biological processes, such as lipid metabolism, which have a significant impact on cancer occurrence and progression, may introduce potential biomarkers for pancreatic cancer screening, as well as new treatment strategies and targets.

Abnormalities in signaling pathways are one of the important advances in cancer. With this in mind, abnormal activity of energy metabolism, such as lipid metabolism, has a unique role in cancer development, and its expression and active state have become of interest to researchers for screening and therapeutic inventions [42, 43]. Based on this, we used a bioinformatics approach to mine the correlation between lipid gene status and prognostic prediction in patients with pancreatic cancer. We found significant aberrant expression of *CEP55*, *CA8*, *GNB3* and *SGSM2* as the key dysregulated metabolic factors within the study population. Overexpression of *CEP55* activates p21 and enhances the cell‐cycle transition. Also, *CEP55* upregulation promotes PANC cell aggressiveness via activating pancreatic cancer [44]. Many studies have shown that *CA8* is associated with poor prognosis in a variety of tumors, [45, 46, 47], but it has not been reported in pancreatic cancer. *GNB3* was reported to influence development of metastasis in low‐grade tumors [48]. *SGSM2* downregulation promoted estrogen receptor‐positive breast cancer cell migration via modulating cell adhesion and cytoskeleton dynamics [49]. These published reports reinforce the potential of these genes as a comprehensive prognosis. We screened prognostic genes from lipid metabolism‐related genes and divided four molecular subtypes to select four genes that are likely to be involved in lipid metabolic processes, although these genes have not been studied in depth in lipid metabolism studies. On this basis, we suggest that four gene signatures are likely to serve as prognostic biological indicators of pancreatic cancer, and that these genes may be involved in important lipid metabolism processes.

Inevitably, there are some limitations in the research, and we hope to address these in future work. First, although three study cohorts were included in this study, our findings should be confirmed in a separate cohort. Second, the prognostic value of lipid metabolism genes was studied using gene microarrays, and this single assay should also be validated by other methods, such as real‐time quantitative RT‐PCR. Third, the majority of genes in our prognostic model have not been reported in studies related to lipid metabolism. Their specific clinical significance, biological function and potential mechanism of action should be studied in further experiments. In conclusion, more experimental evidence is needed to determine the function of these genes in pancreatic cancer.

## Conclusions

Our study found that four lipid metabolism‐related genes were significantly associated with prognosis in patients with pancreatic cancer; therefore, the four‐gene signature with some clinicopathological characteristics could be a useful biomarker for the prognosis of pancreatic cancer.

## Conflict of interest

The authors declare no conflict of interest.

## Author contributions

YY and HW conceived and designed the research. YQ drafted the manuscript and agreed to be accountable for all aspects of the work in ensuring that questions related to the accuracy or integrity of any part of the work are appropriately investigated and resolved. ZC contributed to date acquisition. YS analyzed data. YY interpreted data. HW revised the manuscript for important intellectual content. All authors approved the final version to be published.

## Supporting information


**Fig. S1.** KM prognosis curves of four molecular subtypes. (A) KM curve between C1 and C2 molecular subtypes. (B) KM curve between C1 and C3 molecular subtypes. (C) KM curve between C1 and C4 molecular subtypes. (D) KM curve between C2 and C3 molecular subtypes. (E) KM curve between C2 and C4 molecular subtypes. (F) KM curve between C3 and C4 molecular subtypes.
**Fig. S2.** Comparison of clinical characteristics of molecular subtypes. (A) Sample distribution of T stages in four subtypes. (B) Sample distribution of N stages in four subtypes. (C) Sample distribution of M stages in four subtypes. (D) Sample distribution of TNM stages in four subtypes. (E) Sample distribution of tumor stages in four subtypes. (F) Sample distribution of sex stages in four subtypes. (G) Sample distribution of age stages in four subtypes.
**Fig. S3.** Comparison of immune characteristics in molecular subtypes. (A) B cell score between molecular subtypes. (B) CD4 cell score between molecular subtypes. (C) CD8 cell score between molecular subtypes. (D) Neutrophil cell score between molecular subtypes. (E) Macrophage cell score between molecular subtypes. (F) Dendritic cell score between molecular subtypes. (G) Stromal score between molecular subtypes. (H) Est_Immune score between molecular subtypes. (I) ESTIMATE score between molecular subtypes.
**Fig. S4.** Prognostic ability of four‐gene signature. (A, D) ROC curve and KM curve of RiskScore in GSE28735 dataset. (B, E) ROC curve and KM curve of RiskScore in GSE62452 dataset. (C, F) ROC curve and KM curve of RiskScore in GSE85916 dataset.

## Data Availability

The analyzed datasets generated during the study are available from the corresponding author on reasonable request.
