# Supplementary material for: Development and validation of a four‐lipid metabolism gene signature for diagnosis of pancreatic cancer
Source: FEBS Open Bio. 2021 Sep 20;11(11):3153–70. doi: 10.1002/2211-5463.13074 (PMC8564347; doi:10.1002/2211-5463.13074)
Supplement: Supplementary file 1 — Fig. S1. KM prognosis curves of four molecular subtypes. (A) KM curve between C1 and C2 molecular subtypes. (B) KM curve between C1 and C3 molecular subtypes. (C) KM curve between C1 and C4 molecular subtypes. (D) KM curve between C2 and C3 molecular subtypes. (E) KM curve between C2 and C4 molecular subtypes. (F) KM curve between C3 and C4 molecular subtypes. Fig. S2. Comparison of clinical characteristics of molecular subtypes. (A) Sample distribution of T stages in four subtypes. (B) Sample distribution of N stages in four subtypes. (C) Sample distribution of M stages in four subtypes. (D) Sample distribution of TNM stages in four subtypes. (E) Sample distribution of tumor stages in four subtypes. (F) Sample distribution of sex stages in four subtypes. (G) Sample distribution of age stages in four subtypes. Fig. S3. Comparison of immune characteristics in molecular subtypes. (A) B cell score between molecular subtypes. (B) CD4 cell score between molecular subtypes. (C) CD8 cell score between molecular subtypes. (D) Neutrophil cell score between molecular subtypes. (E) Macrophage cell score between molecular subtypes. (F) Dendritic cell score between molecular subtypes. (G) Stromal score between molecular subtypes. (H) Est_Immune score between molecular subtypes. (I) ESTIMATE score between molecular subtypes. Fig. S4. Prognostic ability of four‐gene signature. (A, D) ROC curve and KM curve of RiskScore in GSE28735 dataset. (B, E) ROC curve and KM curve of RiskScore in GSE62452 dataset. (C, F) ROC curve and KM curve of RiskScore in GSE85916 dataset. [file FEB4-11-3153-s001.zip › feb413074-sup-0005-Legends.docx]

**Supplementary figure legends**

**Figure S1**

KM prognosis curves of four molecular subtypes.

**Figure S2**

**Comparison of clinical characteristics of molecular subtypes.** A: Sample distribution of T stages in four subtypes. B: Sample distribution of N stages in four subtypes. C: Sample distribution of M stages in four subtypes. D: Sample distribution of TNM stages in four subtypes. E: Sample distribution of Stage stages in four subtypes. F: Sample distribution of sex stages in four subtypes. G: Sample distribution of Age stages in four subtypes.

**Figure S3**

**Comparison of immune characteristics in molecular subtypes.** A: B cell score between molecular subtypes. B: CD4 cell score between molecular subtypes. C: CD8 cell score between molecular subtypes. D: Neutrophil cell score between molecular subtypes. E: Macrophage cell score between molecular subtypes. F: Dendritic cell score between molecular subtypes. G: Stromal score between molecular subtypes. H: Est_Immune score between molecular subtypes. I: ESTIMATE score between molecular subtypes.

**Figue S4**

**Verification of gene signature in GSE28735, GSE62452, and GSE85916 dataset.** A: ROC curves in the GSE28735 data set. B: ROC curves in the GSE62452 data set.C: ROC curves in the GSE85916 data set. D: KM curves in the GSE28735 data set for the high-low risk group. E: KM curves in the GSE62452 data set for the high-low risk group. F: KM curves in the GSE28735 data set for high and low risk groups.
